# Assessing the accuracy of artificial intelligence in the diagnosis and management of orbital fractures: Is this the future of surgical decision-making?

**DOI:** 10.1016/j.jpra.2024.09.014

**Published:** 2024-09-30

**Authors:** Steven Gernandt, Romain Aymon, Paolo Scolozzi

**Affiliations:** a Division of Oral and Maxillofacial Surgery, Department of Surgery, Faculty of Medicine, University of Geneva & University Hospitals of Geneva, Geneva, Switzerland; b Division of Oral and Maxillofacial Surgery, Department of Surgery, University of Geneva & University Hospitals of Geneva, Geneva, Switzerland

**Keywords:** Orbital fractures, Artificial intelligence, ChatGPT-4, Decision-making

## Abstract

Orbital fractures are common, but their management remains controversial. The aim of the present study was to assess the accuracy of an advanced artificial intelligence (AI) model, ChatGPT-4, in surgical decision-making, with a focus on orbital fracture diagnosis and management.

A retrospective observational analysis was conducted by involving a sample of 30 orbital fracture cases diagnosed and managed at the Geneva University Hospital, Switzerland. The process involved creating patient vignettes from anonymised medical records and presenting them to ChatGPT-4 in three stages: initial diagnosis, refinement with radiological reports and surgical intervention decisions. The performance of ChatGPT-4 in providing the appropriate surgical strategy was evaluated through measures of sensitivity, specificity, positive predictive value and negative predictive value, with the actual management used as the benchmark for accuracy.

The AI model could correctly diagnose the fracture in 100 % of the cases. It demonstrated a specificity of 100 % and sensitivity of 57 % for treatment recommendation, indicating its effectiveness in recognising patients who truly required an intervention; however, it demonstrated a moderate performance in correctly identifying cases that were better suited for conservative treatment. Cohen's Kappa statistic for interrater reliability of the choice of treatment was 0.44, indicating a weak level of agreement between ChatGPT and the physician's choice of treatment.

The study demonstrates that AI tools such as ChatGPT-4 can offer a high degree of accuracy in diagnosing orbital fractures and recognising patients requiring surgical intervention; however, it performed less satisfactorily in correctly identifying patients who were better suited for non-surgical treatment.

## Introduction

Artificial intelligence (AI), a term first coined by John McCarthy in 1956, refers to the technology that enables computers and machines to simulate human intelligence and problem-solving capabilities.^1^^,^^2^ Over the past several decades, AI has evolved dramatically and has become an integral part of our daily lives. It has been used in numerous applications across diverse domains, from virtual assistants to advanced data analytics and beyond.

An application using AI that has gained significant attention over the last few years is ChatGPT, which was developed by OpenAI and released to the public in November 2022 after four years of training and development. This technology works by learning patterns from large sets of text data and accessing the web for current information, enabling it to provide relevant and accurate information on various topics. The large language model processes the information through a complex neural network, using layers of interconnected nodes or ‘neurons’. These neural networks analyse the input data, recognise patterns and generate appropriate responses.^3^

In the medical setting, ChatGPT-4 can offer recommendations based on medical knowledge, by summarising recent publications and suggesting possible diagnoses based on clinical information and proposing the next steps in treatment management.^4^, ^5^, ^6^, ^7^, ^8^

In surgery, the possible applications of AI extend beyond mere diagnostic tools; they offer patient and resident education, surgical planning, intra-operative assistance and post-operative care.^4^^,^^9^, ^10^, ^11^

Orbital fractures are the most common type of facial fracture seen in maxillofacial surgery, with a reported incidence of up to 90 % and representing up to 3 % of all emergency room consults.^12^^,^^13^

Most of these fractures do not result in long-term aesthetic or functional deficits and can be managed without surgery.^14^^,^^15^ Most orbital fractures, except those with specific complications such as trapdoor fractures and disfiguring asymmetries, lack universally accepted treatments.^16^, ^17^, ^18^ Although the debate about non-surgical repair continues, there has been a recent trend towards more conservative management and significant reduction in surgical interventions.^16^, ^17^, ^18^

The proliferation of publications in recent years offering ideal decision-making guidelines indicates that this issue is unresolved.^19^, ^20^, ^21^, ^22^, ^23^, ^24^ Moreover, the vast and constantly evolving body of knowledge makes it difficult to apply these guidelines effectively in daily practice.^19^, ^20^, ^21^, ^22^, ^23^, ^24^ Therefore, there is a clear opportunity to apply AI in this area. AI can quickly provide real-time insights based on a summary of current trends, a task that would be extremely time-consuming for a human to perform manually.

Studies exploring the use of ChatGPT in medical diagnostics and management continue to emerge in the literature. To date, existing publications have primarily focused on the use of AI for ‘technical’ purposes, such as segmentation and measurement of orbital fracture areas.^25^, ^26^, ^27^ To our knowledge, no studies have investigated the role of AI in the diagnosis and management of orbital fractures. The purpose of the present study was to assess the accuracy of ChatGPT-4 in the diagnosis and management of orbital fractures compared to the actual clinical decisions made in our department.

## Material and methods

### Study design and sample

To address this research purpose, the investigators designed and implemented a retrospective observational study. The present study followed the Declaration of Helsinki guidelines and was approved by the ethics commission of the University Hospitals of Geneva.

The study population was selected from a database of patients who were diagnosed with orbital fractures and treated in the Oral and Maxillofacial Surgery division at the Geneva University Hospital, Switzerland, between 2009 and 2024.

Only patients with a diagnosis of orbital fractures confirmed by clinical examination and diagnostic imaging and with complete medical records were included in this study. Thirty cases were selected to best represent the diversity of orbital fractures. This included a wide range of patient ages, pure and impure fracture types, variations in fracture size and incarceration and non-incarceration of periorbital tissues and the inferior rectus muscle (IRM). All data were stored on REDCap (research electronic data capture) database, a password-protected, secure data collection and storage web application.

The initial charting data were extracted for each patient and included the chief complaint, mechanism of injury, past medical history when relevant and results of the clinical examination. Radiological report data were also extracted. These data were then converted into the format of a standardised vignette and included:

- Age and gender of patient
- Mechanism of injury
- Current medical history pertaining to the event
- Relevant past medical history and comorbidities
- Physical examination findings
- Diagnostic imagery findings

The purpose of the vignette was to imagine how the case would be presented to the consultant or senior/supervising physician.

### Example

a) *As documented:*

A 57-year-old patient, known for treated hypertension, fell today after experiencing dizziness resulting in trauma to the right eye against a piece of furniture. There is a lower eyelid laceration. No decrease in visual acuity or diplopia was noted. The patient has a known allergy to penicillin.

### Clinical examination

• Dermabrasion above the right eyebrow
• Right lower eyelid superficial laceration, 3.5 cm long, without the involvement of the free edge, starting from the outer corner then descending to the top of the cheekbone.
• Periorbital hematoma
• Painful palpation of the orbital rim without step deformity
• Pupils are equal, round and reactive to light; no relative afferent pupillary defect
• Free, painless ocular motility without diplopia
• No change to visual acuity
• Normal facial sensitivity/motility

b) *Vignette version:*

A 57-year-old patient with a history of controlled hypertension presents to the emergency department after a fall induced by dizziness, resulting in right-sided facial trauma against an item of furniture. The patient reported an injury to the right eye but denied any decrease in visual acuity or experiencing double vision. Notably, the patient has a penicillin allergy.

On examination, you observe dermabrasion above the right eyebrow and a superficial 3.5-cm long laceration on the right lower eyelid, extending from the outer corner to the top of the cheekbone. A periorbital hematoma is present. Palpation of the orbital rim is painful, but there is no step deformity. The pupils are equal, round and reactive to light, with no signs of a relative afferent pupillary defect. Ocular motility is unrestricted and painless in all directions, without any diplopia. Visual acuity is preserved. Facial sensitivity and motility are intact.

Following the initial assessment, a CT scan was performed to evaluate the extent of the facial trauma. The scan findings are as follows:

• No intra-cranial bleeding detected
• Blow-out fracture of the right orbital floor, affecting the right infra-orbital nerve canal, with herniation of the IRM
• Presence of extraconal and subcutaneous emphysema
• Hematoma in the right maxillary sinus
• A probable colloid cyst in the third ventricle

The vignettes of the 30 patients were then presented to ChatGPT-4 (launched April 2023, updated April 2024 Version, OpenAI, San Francisco, USA), which was interrogated regarding the diagnosis and possible treatment strategies. Further information such as radiological findings, diagnosis and suggested management were collected but withheld from the vignette.

For consistency, the entire procedure was carried out by a single investigator (SG) who entered the data and compared the diagnostic-therapeutic suggestions of ChatGPT-4 with the actual decisions.

Notably, the decision for surgical repair was based on our in-house clinical protocol as follows^13^: 1) immediate restriction of ocular motility to vertical gaze in at least one visual field and/or primary gaze diplopia that warranted urgent treatment or bothersome diplopia (double vision that interferes with the normal performance of common daily tasks such as reading, walking and driving) that did not resolve at the 10-day follow-up and 2) enophthalmos that was immediately apparent to the naked eye and persisted until the 10-day follow-up.

### Chat GPT4 input

Given the sensitivity of the medical records, particular care was taken to the anonymise the data. Once reformatted into a vignette, they were reviewed independently by two reviewers (SG) and (PS) to check for any possible identifying information.

1. The first stage of the process involved using the following prompt:
  - ‘ChatGTP, imagine you are a maxillofacial surgeon and are presented with the following case (Insert vignette X), what would be your list of three differential diagnoses, with arguments and what additional tests would you require to find the correct diagnosis?’
2. The second stage of the process involved proving ChatGPT with the radiological report findings.
  - ‘After the initial assessment, a CT scan was performed, these are the findings: (Insert radiology report X). What is the most probable diagnosis and what are the next steps in management, with reasons?’
3. The third stage involved asking ChatGPT to decide to offer surgery or not and if needed which approach and technique it would use.
  - ‘In this case, you need to decide whether to offer surgery or not. If you do suggest surgery, what surgical approach and technique would you use and why? Is there any degree of urgency?’

### Data analysis

All statistical analysis were performed using the R statistical software (version 4.3.3; R Development Core Team, Vienna, Austria). Patient characteristics were summarised using descriptive statistics, with mean and standard deviation reported for normally distributed continuous variables and counts and percentages reported for categorical variables. Owing to small cell counts, we used the Fisher's exact test to analyse bivariate associations between the categorical variables. The performance of ChatGPT-4 in providing the appropriate surgical strategy was evaluated through measures of sensitivity, specificity, positive predictive value (PPV) and negative predictive value (NPV), with the actual management as the benchmark for accuracy. The interrater reliability between the treatment recommendation given by ChatGPT-4 and actual surgery was measured using the Cohen's Kappa statistic. Significance level was set at *P* ≤ 0.05.

## Results

A total of 30 cases of orbital fractures diagnosed between 2009 and 2024 at the Geneva University Hospital, Switzerland, were included in this study. Descriptive statistics are presented in Table 1. The mean age at the time of trauma was 34.1 ± 22.8 years, with a male predominance (*n* *=* *21*; 70 %). The most common aetiology was sports-related accidents (*n* *=* *9;* 30 %), followed by assault (*n* *=* *8;* 27 %). Most fractures (*n* *=* *22*; 73 %) were pure orbital fractures, without IRM herniation (*n* *=* *20*; 66.7 %) and were treated conservatively (*n* *=* *18*, 60 %).

**Table 1:** General characteristics.

	Patients (*n* = 30)
Age (y) (mean [SD])	34.1 (22.8)
Range (y)	5–81
Sex (%)	
F	9 (30.0)
M	21 (70.0)
Aetiology (%)	
Assault	8 (26.7)
Fall	7 (23.3)
Sport accident	9 (30.0)
Traffic accident	6 (20.0)
Incarceration of ocular muscles (%)	
No	20 (66.7)
Yes	10 (33.3)
Treatment (%)	
Surgery	12 (40.0)
Conservative	18 (60.0)
Fracture type (%)	
Pure orbital	22 (73.3)
Impure orbital	8 (26.7)

Based on the given vignette, ChatGPT-4 correctly diagnosed the fracture in 100 % of cases and suggested appropriate next steps in management (Table 2). However, there was a certain discrepancy between the choice of conservative versus surgical treatment with an overall mismatch of 30 %. ChatGPT-4 demonstrated a specificity of 100 %, indicating its effectiveness in identifying patients who truly needed surgery and a sensitivity of 57 %, indicating moderate performance in correctly identifying cases that were better suited for conservative management. The associated PPV and NPV are 100 % and 50 %, respectively, suggesting that although the model perfectly identified all patients requiring surgery, it correctly identifies only half of the patients who would benefit from a conservative approach. Cohen's Kappa statistic for interrater reliability of choice of treatment was 0.44, indicating a weak level of agreement between the ChatGPT-4 and physician's actual choice of treatment. There was a greater rate of concordance in the management of impure fractures compared to pure fractures (62.5% vs 72.7 %) and fractures that involved the incarceration of IRM and periorbital tissues (80% vs 65 %).

**Table 2:** Concordance of ChatGPT4 vs actual diagnosis and management.

	Conservative (*n* = 18)	Surgery (*n* = 12)	*P*
**Diagnosis (GPT)**			
Concordance	18 (100.0)	12 (100.0)	***
No concordance	0 (0.0)	0 (0.0)	
**Treatment suggested (GPT)**			
Concordance	9 (50.0)	12 (100.0)	<0.01
No concordance	9 (50.0)	0 (0.0)	
	Pure (*n* = 22)	Impure (*n* = 8)	*P*

**Diagnosis (GPT)**			
Concordance	22 (100.0)	8 (100.0)	***
No concordance	0 (0.0)	0 (0.0)	
**Treatment suggested (GPT)**			
Concordance	16 (72.7)	5 (62.5)	0.67
No concordance	6 (27.3)	3 (37.5)	
	Incarceration (*n* = 10)	N/incarceration (*n* = 20)	*P*

**Diagnosis**			
Concordance	10 (100.0)	20 (100.0)	***
No concordance	0 (0)	0 (0)	
**Treatment suggested**			
Concordance	8 (80.0)	13 (65.0)	0.67
No concordance	2 (20.0)	7 (35.0)	

^⁎⁎⁎^ The *P* value cannot be calculated due to the 100% agreement between the two groups.

## Discussion

This retrospective observational study described and analysed the data from 30 cases of orbital fractures in terms of actual diagnosis and management and compared it to the suggested diagnosis and management offered by ChatGPT-4.

The author's hypothesis was that the large language model would provide an accurate diagnosis when given sufficient clinical and radiological data and would suggest relevant and reasonable next steps in management for most cases; however, given the controversies in the management of these fractures, it was expected to display some degree of discrepancy.

The results of the present study have substantially confirmed our hypothesis. First, the results show that ChatGPT-4 can accurately output the diagnosis in all cases. During the first stage of the process, ChatGPT-4 was queried to offer three differential diagnoses based on the medical history, clinical presentation and mechanism of injury. While periorbital swelling, ecchymosis and diplopia are often highly suggestive of an orbital fracture especially when considering the mechanism, the AI model generated three plausible differentials in order of probability, providing relevant and accurate arguments for each. It successfully identified orbital fracture as the most likely diagnosis in all but one case, where the condition was highly suggestive of a neurosurgical emergency and ChatGPT-4 generalised the third differential diagnosis as ‘possible facial fractures’.

If no information was provided initially, the AI model would always suggest additional exams, including CT-imaging of the facial skeleton and, in cases of high velocity trauma or if any neurological symptoms, a CT scan for detecting intracranial haemorrhage or MRI to assess a traumatic brain injury.

The radiology reports were input into ChatGPT-4 during the second stage of the process, and this allowed the model to provide a more accurate diagnosis and suggest next stages of management.

The final stage of the process involved querying the AI model to decide if surgical intervention is required. Based on our results, ChatGPT-4 was accurate in recognising patients who required surgical intervention but could only identify 57 % of patients who could have been treated nonsurgically. This indicates that AI is more aggressive with management and suggests surgical interventions more often than in actual clinical practice. A possible explanation for this relates to how ChatGPT-4 functions, and the source of the information it uses. On searching the web or scientific publications on orbital fractures, conflicting information is retrieved. Moreover, there is a tendency to offer surgery to prevent poor aesthetic and functional outcomes; however, no strict consensual guidelines currently exist. At the Geneva University Hospital, the decision on whether to treat the patient conservatively is based on a set of in-house criteria that consider bothersome diplopia at the baseline and progression, enophtalmia and ultimately patient-related characteristics.^13^ We purposely did not provide the AI model with our criteria as to not create a bias.

ChatGPT-4 appeared to be rather consistent in suggesting surgical management of large fractures, except in one case where the patient presented with a large orbital floor fracture (>3 cm^2^), with hypotropia and discrete enophtalmia. ChatGPT-4 suggested conservative treatment, which was a surprising as the literature often mentions that a surface area >2 cm^2^ as the agreed cut-off for surgery as it leads to a greatest risk of enophthalmos.^16^^,^^18^ In practice, this case was managed conservatively with a successful outcome. When asked why nonsurgical treatment was suggested, despite the large surface area of the fracture and discrete enophtalmia, the model reformulated its proposition to initially offer nonsurgical management, but indicated that close follow-up should be provided with the possibility of a surgical intervention if bothersome diplopia did appear or for aesthetic concerns in relation to enophtalmia.

During our preliminary tests, especially when testing the model with more extreme cases, such as a paediatric white-eye blow-out fracture with periorbital tissue entrapment and neurovegetative symptoms (i.e. nausea and vomiting), the AI model strongly suggested surgical management, without any mention of urgency. It was only after modifying the initial prompt to include the degree of urgency into the management that the output was clear that urgent surgery would be required. At present, this technology does not have to the ability to think for us and provide unsolicited information, but it aims to complete the task set and provide an answer to the questions asked.

An interesting aspect of using AI in this way is that it aids in decision making and offers significant educational benefits for trainee doctors by providing clear explanations to its answers.

A realisation, when reading the current literature on the use of ChatGPT, was the lack of transparency with regards to the specific prompts used in the methodology of the various publications.^4^, ^5^, ^6^, ^7^, ^8^, ^9^ As mentioned previously, there is a huge importance related to the prompt as it greatly affects the desired output and accuracy of the response.

While the technology continues to improve and becomes more powerful, the accuracy of the information provided should always be evaluated carefully as it can, at times, ‘hallucinate’ and provide plausible sounding, yet false and nonsensical responses. Without prior knowledge or critical analysis of the suggestions, the risk of relying of GPT-4 alone, for decision making could lead to serious consequences.^4^, ^5^, ^6^, ^7^, ^8^, ^9^

The main strength of this study is that it is the first article to explore the use of AI in the diagnosis and management of orbital fractures. This research contributes significantly to the emerging literature on AI applications in specialised medical fields.

However, the data generated in the present study should be interpreted in the context of certain limitations: first, the retrospective, single-institution design; second, the small sample size with an insufficient number of patients to perform multivariate analysis and third, the AI model, is guided by the user inputs and prompts, while careful phrasing was used, it is likely that the results could be altered by making slight changes to the prompt given to ChatGPT.

Future studies should focus on comparing ChatGPT-4 with other AI tools, such as Google's Gemini Pro, for surgical decision support. To date, only one study has evaluated AI in plastic and reconstructive surgery, demonstrating that although ChatGPT-4 generally provides more medically accurate and relevant responses than Gemini, both models have sufficient knowledge to effectively assist surgeons during procedures.^28^

## Conclusion

In conclusion, the study demonstrated that AI tools such as ChatGPT-4 can offer a high degree of accuracy and efficiency and have the potential to be used as tools for assisted decision-making. However, these results are based on a small sample size and are a proof-of-concept that merits further exploration and investigation.

An objective of our future research is to conceptualise an AI-integrated system that can rapidly process patient data to provide a probabilistic differential diagnosis and management pathway.

## Ethical approval

The study was conducted in accordance with the Declaration of Helsinki and was approved by our local ethical board (Study number 12–255).

## Funding

None.

## Conflict of interest

None.

## References

[1] Anyoha R. The history of artificial intelligence. Artificial intelligence: A modern approach. 2017. https://sitn.hms.harvard.edu/flash/2017/history-artificial-intelligence/.

[2] Amisha M.P, Pathania M., Rathaur V.K (2019). Overview of artificial intelligence in medicine. J Fam Med Prim Care.

[3] Briganti G (2024). How ChatGPT works: A mini review. Eur Arch Otorhinolaryngol.

[4] Mintz Y., Brodie R (2019). Introduction to artificial intelligence in medicine. Minim Invasive Ther Allied Technol.

[5] Truhn D., Weber C.D., Braun B.J., Bressem K., Kather J.N., Kuhl C. (2023). A pilot study on the efficacy of GPT-4 in providing orthopedic treatment recommendations from MRI reports. Sci Rep.

[6] Lee R.Y., Ziehm A.I., Ullrich L., Stawicki S.P, Stawicki PS (2023). Artificial Intelligence.

[7] Pagano S., Holzapfel S., Kappenschneider T., Meyer M., Maderbacher G., Grifka J. (2023). Arthrosisdiagnosis and treatment recommendations in clinical practice: An exploratory investigation with the generative AI model GPT-4. J Orthop Traumatol.

[8] Mira F.A., Favier V., Dos Santos Sobreira Nunes H., De Castro J.V., Carsuzaa F., Meccariello G. (2024). Chat GPT for the management of obstructive sleep apnea: Do we have a polar star?. Eur Arch Otorhinolaryngol.

[9] Hassan A.M., Nelson J.A., Coert J.H., Mehrara B.J., Selber J.C (2023). Exploring the potential of artificial intelligence in surgery: Insights from a conversation with ChatGPT. Ann Surg Oncol.

[10] Malhotra K., Wong B.N.X., Lee S., Franco H., Singh C., Cabrera Silva L.A. (2023). Role of artificial intelligence in global surgery: A review of opportunities and challenges. Cureus.

[11] Pakkasjärvi N., Luthra T., Anand S (2023). Artificial intelligence in surgical learning. Surgeries.

[12] Bord S.P., Linden J (2008). Trauma to the Globe and Orbit. Emerg Med Clin North Am.

[13] Scolozzi P (2022). Reflections on a patient-centered approach to treatment of blow-out fractures: Why the wisdom of the pastmust guide our decision-making. J Plast Reconstr Aesthet Surg.

[14] Lynham A.J., Chapman P.J., Monsour F.N., Snape L., Courtney D.J., Heggie A.A. (2004). Management of isolated orbital floor blow-out fractures: A survey of Australian and New Zealand oral and maxillofacial surgeons. Clin Experiment Ophthalmol.

[15] Putterman A.M., Stevens T., Urist M.J (1974). Nonsurgical management of blow-out fractures of the orbital floor. Am J Ophthalmol.

[16] Zhou P., Chambers C.B (2021). Orbital fractures. Semin Plast Surg.

[17] Billig A.B., Dengler J., Hardisty M., Chew H.F., Kiss A., Fialkov J.A (2023). Are we overoperating on isolated orbital floor fractures?. Plast Reconstr Surg.

[18] Dubois L., Steenen S.A., Gooris P.J., Mourits M.P., Becking A.G (2015). Controversies in orbital reconstruction—I. Defect-driven orbital reconstruction: A systematic review. Int J Oral Maxillofac Surg.

[19] Dubois L., Steenen S.A., Gooris P.J., Mourits M.P., Becking A.G (2015). Controversies in orbital reconstruction–II. Timing of post-traumatic orbital reconstruction: A systematic review. Int J Oral Maxillofac Surg.

[20] Dubois L., Steenen S.A., Gooris P.J., Bos R.R., Becking A.G (2016). Controversies in orbital reconstruction-III. Biomaterials for orbital reconstruction: A review with clinical recommendations. Int J Oral Maxillofac Surg.

[21] Alinasab B., Borstedt K.J., Rudstrom R., Ryott M., Qureshi A.R., Beckman M.O. (2018). New algorithm for the management of orbital blowout fracture based on prospective study. Craniomaxillofac Trauma Reconstr.

[22] Alinasab B., Borstedt K.J., Rudstrom R., Ryott M., Qureshi A.R., Stjärne P (2018). Prospective randomized controlled pilot study on orbital blowout fracture. Craniomaxillofac Trauma Reconstr.

[23] Frohwitter G., Wimmer S., Goetz C., Weitz J., Ulbig M., Kortuem K.U. (2018). Evaluation of a computed-tomography-based assessment scheme in treatment decision-making for isolated orbital floor fractures. J Craniomaxillofac Surg.

[24] De Ruiter B.J., Kotha V.S., Lalezar F.D., Swanson M.A., Kumar A.R., Barmettler A. (2022). Orbital index: A novel comprehensive quantitative tool for prediction of delayed enophthalmos in orbital floor fracture management. Plast Reconstr Surg.

[25] Morita D., Kawarazaki A., Koimizu J., Tsujiko S., Soufi M., Otake Y. (2023). Automatic orbital segmentation using deep learning-based 2D U-net and accuracy evaluation: A retrospective study. J Craniomaxillofac Surg.

[26] Kang D (2023). Evaluating the accuracy and reliability of blowout fracture area measurement methods: A review and the potential role of artificial intelligence. J Craniofac Surg.

[27] Bao X.L., Zhan X., Wang L., Zhu Q., Fan B., Li G.Y (2023). Automatic identification and segmentation of orbital blowout fractures based on artificial intelligence. Transl Vis Sci Technol.

[28] Gomez-Cabello C.A., Borna S., Pressman S.M., Haider S.A., Forte A.J (2024). Large language models for intraoperative decision support in plastic surgery: A comparison between ChatGPT-4 and Gemini. Medicina (B Aires).

